# Enzymatic Resistance of Corneas Crosslinked Using Riboflavin in Conjunction With Low Energy, High Energy, and Pulsed UVA Irradiation Modes

**DOI:** 10.1167/iovs.15-18769

**Published:** 2016-04-05

**Authors:** Nada H. Aldahlawi, Sally Hayes, David P. S. O'Brart, Alina Akhbanbetova, Stacy L. Littlechild, Keith M. Meek

**Affiliations:** 1 Structural Biophysics Research Group, School of Optometry and Vision Sciences, Cardiff University, Cardiff, United Kingdom; 2 Cardiff Institute for Tissue Engineering and Repair (CITER), Cardiff University, Cardiff, United Kingdom; 3 Keratoconus Research Institute, Department of Ophthalmology, St. Thomas' Hospital, London, United Kingdom

**Keywords:** keratoconus, crosslinking, accelerated crosslinking, CXL, enzymatic digestion

## Abstract

**Purpose:**

To investigate the effect of various riboflavin/ultraviolet light (UVA) crosslinking (CXL) protocols on corneal enzymatic resistance.

**Methods:**

A total of 66 enucleated porcine eyes, with the corneal epithelium removed, were divided into 6 groups. Group 1 remained untreated. Groups 2 to 6 received riboflavin/dextran for 30 minutes. Group 3 underwent standard CXL (SCXL) with 3 mW/cm^2^ UVA for 30 minutes (total energy dose 5.4 J/cm^2^). Groups 4 and 5 underwent high intensity CXL (HCXL) using 30 mW/cm^2^ UVA for 3 minutes (5.4 J/cm^2^) and 30 mW/cm^2^ for 4 minutes (7.2 J/cm^2^), respectively. Group 6 was exposed to 8 minutes of 30 mW/cm^2^ UVA in a 10-second on/10-second off pulsed-radiation mode (p-HCXL; 7.2 J/cm^2^). A central 8-mm disk from each cornea was submerged in pepsin digest solution at 23°C and measured daily. After 13 days, the dry weight was recorded from 5 samples in each group.

**Results:**

The CXL-treated corneas took longer to digest than nonirradiated corneas (*P* < 0.0001). Differences in digestion time also were observed between CXL groups, such that, HCXL (5.4 J/cm^2^) < SCXL (5.4 J/cm^2^) < HCXL (7.2 J/cm^2^) < p-HCXL (7.2 J/cm^2^; *P* < 0.0001). The dry weight of the SCXL (5.4 J/cm^2^) group was higher than the HCXL (5.4 and 7.2 J/cm^2^; *P* < 0.001) and p-HCXL 7.2 J/cm^2^ (*P* <0.05) groups. No difference was detected between the HCXL and p-HCXL 7.2 J/cm^2^ groups.

**Conclusions:**

The intensity and distribution of the crosslinks formed within the cornea vary with different UVA protocols. The precise location and amount of crosslinking needed to prevent disease progression is unknown.

Keratoconus is a degenerative corneal dystrophy, characterized by progressive corneal thinning and subsequent impairment of corneal biomechanics.^1–3^ The resultant conical ectasia causes irregular astigmatism and associated reduction of visual performance, which can be significant.^4^ It typically presents in adolescence and is the most common of all corneal dystrophies with a reported incidence of 1 in 1750.^1–3^ Its precise pathophysiology is as yet undetermined, but it has been shown to be associated with an upregulation of degradative proteolytic enzymes.^5^ Riboflavin and ultraviolet A (UVA) corneal crosslinking (CXL) was first postulated in 1998 as a means of strengthening the corneal stroma, increasing its resistance to enzymatic digestion and stabilizing cases of progressive keratoconus.^6^ It has since been the subject of a plethora of research articles investigating and confirming its safety and efficacy.^7–10^

The standard CXL protocol (SCXL), first clinically tested by Wollensak et al.,^8^ involves removing the central 9 mm of corneal epithelium, soaking the exposed stromal surface with 0.1% riboflavin for 30 minutes, and irradiating the riboflavin-laden stroma with 370 nm UVA light with an intensity of 3 mW/cm^2^ (resulting in a cumulative dose of 5.4 J/cm^2^). This protocol requires in excess of 1 hour of treatment time. Given its frequency of occurrence, the potential numbers of patients with progressive keratoconus requiring CXL is large and represents a not inconsiderable burden to health services. In an attempt to reduce the treatment time, a number of modifications to the existing SCXL protocol have been proposed. These changes are based primarily on current understanding of the photochemical kinetics of UVA exposure and the theoretical principles of the Bunsen–Roscoe law of reciprocity,^11^ which states that a certain biological effect is directly proportional to the total energy dose irrespective of the administered regimen. However, as has been shown with other photochemical reactions,^12^ this law may only be valid within a certain dose range and this must be defined individually for each reaction.

The precise photochemical mechanism involved in riboflavin/UVA CXL currently is uncertain. What has been shown, however, is that oxygen is essential to drive the process and in the absence of oxygen, CXL is impaired.^13^ It has been suggested that higher intensity UVA, or so-called “accelerated” CXL protocols, result in more rapid oxygen depletion thereby reducing efficacy.^14,15^ It has been shown that by ceasing UV irradiation, oxygen can be restored to its normal level within 3 to 4 minutes^16^ and, on this basis, it has been proposed that by pulsing the UV light throughout the procedure, oxygen levels may be replenished so that the CXL process is no longer impaired.^17,18^ In addition to pulsing, it also has been suggested that the efficacy of accelerated protocols may be improved by increasing the exposure time by 30% to 40%.^19,20^

To further investigate these issues and compare the efficacy of accelerated CXL, extended accelerated CXL and pulsed CXL protocols with the “gold standard” SCXL protocol, we analyzed the rate of enzymatic digestion after CXL in an ex vivo porcine model.

## Methods

### Study Design

A total of 66 fresh porcine eyes, with transparent corneas and an intact corneal epithelium, were obtained from a local European Community licensed abattoir and used within 6 hours of death. Following a complete debridement of the corneal epithelium using a single-edged razor blade, the eyes were divided randomly and equally into the 6 groups described below (in which Groups 1 and 2 served as controls).

1. Untreated (U): no treatment performed.
2. Riboflavin only (R): a 0.1% riboflavin solution containing 20% dextran T-500 (Mediocross D; Peschke Meditrade, Huenenberg, Switzerland) was applied to the anterior corneal surface for 30 minutes using an annular suction ring.
3. Standard low-intensity CXL (SCXL 5.4 J/cm^2^): a 0.1% riboflavin solution containing 20% dextran T-500 was applied to the anterior corneal surface for 30 minutes (as above). The cornea then was exposed to 3 mW UVA for 30 minutes (total energy dose of 5.4 J/cm^2^) during which time riboflavin was reapplied at 5-minute intervals.
4. High intensity 30 mW/3 min CXL (HCXL 5.4 J/cm^2^): a 0.1% riboflavin solution containing 20% dextran T-500 was applied to the anterior corneal surface for 30 minutes. The cornea then was exposed to 30 mW UVA for a period of 3 minutes (total energy dose of 5.4 J/cm^2^) during which time riboflavin was reapplied once.
5. High intensity 30 mW/4 min CXL (HCXL 7.2J/cm^2^): a 0.1% riboflavin solution containing 20% dextran T-500 was applied to the anterior corneal surface for 30 minutes. The cornea then was exposed to 30 mW UVA for a period of 4 minutes (total energy dose of 7.2 J/cm^2^) during which time riboflavin was reapplied once.
6. High intensity 30 mW/8min pulsed CXL (p-HCXL7.2 J/cm^2^): a 0.1% riboflavin solution containing 20% dextran T-500 was applied to the anterior corneal surface for 30 minutes. The cornea then was exposed to 30 mW UVA in a pulsed radiation mode of 10 seconds on and 10 seconds off for a period of 8 minutes (total energy dose of 7.2 J/cm^2^). During irradiation the riboflavin solution was reapplied twice.

All of the irradiation protocols were performed using the Phoenix CXL System (Peschke Meditrade) with a wavelength of 365 nm, a 50 mm working distance, and a 9 mm aperture. Central corneal thickness was measured via ultrasound pachymetry (DGH Pachmate 55; DGH Technologies, Exton, PA, USA) before treatment (after epithelial debridement), after riboflavin application, and after UVA exposure.

Following treatment, an 8 mm full-tissue-thickness biopsy was trephined from the center of each cornea. The corneal disks were placed in individual sealed tubes containing 5 mL pepsin digest solution (1 g 600 to 1200 U/mg pepsin from porcine gastric mucosa [Sigma-Aldrich, Dorset, UK] in 10 mL 0.1 M HCL at pH 1.2) and incubated in a water bath at 23°C. As our previous studies suggest that CXL causes the formation of crosslinks not only at the collagen fibril surface but also in the protein network surrounding the collagen,^21^ pepsin was selected in preference to collagenase as the enzyme of choice for this study.

Using electronic digital calipers, the diameter of the anterior surface of each corneal disk was recorded daily until the tissue could no longer be distinguished from the surrounding pepsin solution (even under microscopic examination). At this point the tissue was considered to have undergone “complete digestion.” We recorded the daily diameter of the trephined corneal disks, rather than corneal thickness, as corneal disks placed in pepsin are known to undergo significant stromal swelling (predominantly in the posterior stroma) during the first 24 hours.^22^

To assess further the effect of each treatment on enzymatic resistance, 5 corneal disks from each group were removed from the pepsin digest solution after 13 days and placed in a 60°C oven until a constant dry weight was obtained. The average corneal dry weight then was calculated for each group.

The corneal disk diameter measurements provide valuable information about the structural integrity of the most anterior layers of the cornea, while the dry weight measurements, representing the total mass of undigested tissue, negate the complications associated with within-sample variations in corneal thickness and between sample differences in hydration and provide information about the effective depth of CXL.

### Data Analysis

Data are shown as mean measurements (±SD) for corneal thickness, dry weight, and complete digestion time. Measurements of corneal disk diameter are presented as a daily cumulative measurement for each treatment group. Statistical analysis was performed using a one-way ANOVA and Bonferroni multiple comparisons in a depth-wise manner. All statistical analyses were performed with the Statistical Package for the Social Sciences (SPSS Statistics 20; IBM, Armonk, NY, USA). A probability value of *P* < 0.05 was considered significant.

## Results

### Corneal Thickness

**Figure 1:**
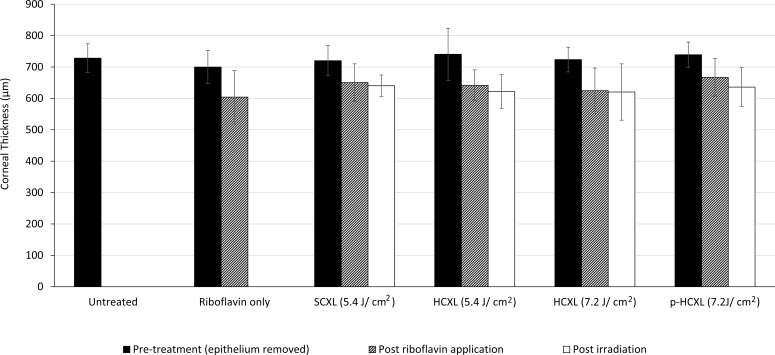
Average corneal thickness measured before, during, and after treatment.

The average stromal thickness at each stage of treatment is shown in Figure 1. Before treatment, the average stromal thickness did not differ significantly between groups. However, a 30-minute application of riboflavin–dextran solution (groups 2–6) resulted in a significant decrease in stromal thickness (*P* < 0.0001). The subsequent irradiation of corneas in groups 3 to 6 produced no further changes in corneal thickness and the final stromal thickness did not differ significantly between any of the CXL groups.

### Time Taken for Complete Digestion

**Figure 2:**
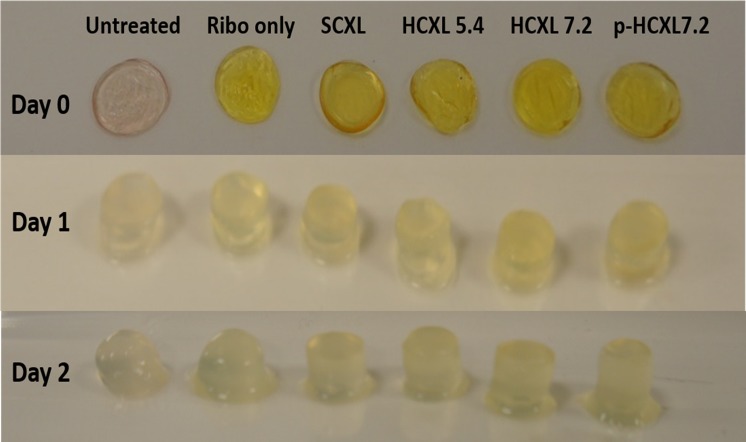
Photographs of a representative corneal disk from each treatment group before immersion in pepsin digest solution (day 0) and after 1 and 2 days of digestion.

Stromal swelling, in a posterior–anterior direction was observed in all corneal disks within 1 day of submersion in pepsin digest solution (Fig. 2). After 2 days of digestion, a loss of structural integrity was seen in the untreated corneas but the crosslinked corneas remained intact (Fig. 2). By day 7 of the digestion process, the anterior portion of each treated and untreated corneal button had separated from the posterior portion and by day 10, the posterior portion had been completely digested in all cases. The anterior corneal disk persisted considerably longer (particularly in the CXL-treated corneas) and maintained its form sufficiently to allow reliable measurements of corneal disk diameter to be recorded daily.

**Figure 3:**
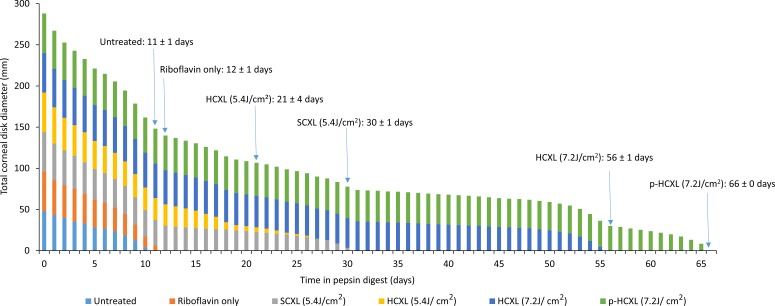
The summed diameter of all corneal disks (*n* = 6) within each crosslinked and noncrosslinked treatment group is shown as a function of time in pepsin digest solution. In addition, the average time (±SD) required for complete digestion of each treatment group has been added to the time-line.

The time required for complete digestion of the crosslinked corneas (groups 3–6) was significantly longer than that required for the nonirradiated specimens (groups 1 and 2; *P* < 0.0001; Fig. 3). After 13 days of digestion, all nonirradiated corneas had undergone complete digestion and the average diameter of all the crosslinked corneal disks had decreased in diameter from their original value.

Corneas crosslinked with higher energy dose treatments (7.2 J/cm^2^) using continuous (group 5) or pulsed (group 6) light took significantly longer to digest than corneas crosslinked using lower (5.4J/cm^2^) energy dose treatments (groups 3 and 4; *P* < 0.0001). A direct comparison between treatments using the same energy dose revealed that corneas crosslinked using the SCXL (5.4 J/cm^2^) procedure took longer to digest than corneas crosslinked using the accelerated HCXL (5.4 J/cm^2^) procedure (*P* < 0.0001), and corneas crosslinked using the pulsed irradiation p-HCXL (7.2 J/cm^2^) procedure took significantly longer to digest than those treated with the continuous irradiation HCXL (7.2J/cm^2^) procedure (*P* < 0.0001).

### Undigested Tissue Mass

**Figure 4:**
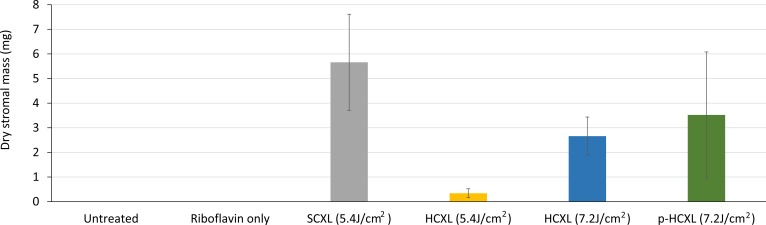
Corneal disk dry weight after 13 days of digestion.

After 13 days in pepsin digest solution, only the CXL-treated corneas remained (Fig. 4). At this time point, the average stromal dry weight of the SCXL (5.4 J/cm^2^)–treated corneas was significantly higher than that of the HCXL 5.4 J/cm^2^– (*P* < 0.0001), HCXL 7.2 J/cm^2^– (*P* < 0.001), and p-HCXL 7.2J/cm^2^– treated corneas (*P* < 0.05). The stromal dry weight did not differ significantly between the two higher energy treatment groups which used continuous (HCXL 7.2 J/cm^2^) and pulsed (p-HCXL 7.2 J/cm^2^) irradiation, but the corneas treated with p-HCXL 7.2 J/cm^2^ had a higher stromal dry weight than the corneas treated with HCXL 5.4 J/cm^2^ (*P* < 0.0001).

## Discussion

Crosslinking has been shown to be a safe and effective treatment for keratoconus^7–10^ and other corneal ectatic disorders.^23,24^ The efficacy of CXL can be attributed, at least in part, to its ability to increase the enzymatic resistance of corneal tissue, as enzymatic digestion is known to be involved in the pathogenesis of keratoconus.^25^

An increase in enzymatic resistance following CXL with irradiances of 2 and 3 mW/cm^2^ UVA was first evidenced by Spoerl et al.^22^ and later by others.^21^ It since has been shown that the use of CXL with irradiances of 9 and 18 mW/cm^2^ also results in enhanced enzymatic resistance.^26^ However measurements of undigested tissue mass midway through the digestion process revealed significant differences between treatment groups that indicated that the amount of CXL may be less when higher intensity “accelerated” protocols, with the same cumulative dose as SCXL, are used.^26^ While some studies show little differences between SCXL and accelerated CXL, a possible reduction in efficacy with high-intensity UVA protocols has been documented recently by some clinical investigators. Ng et al.^27^ compared 9 mW/cm^2^ for 10 minutes to SCXL and reported a statistically greater reduction in maximum and mean keratometry and deeper demarcation lines in SCXL-treated eyes.^27^ Similar results were reported in a retrospective study by Brittingham et al.^14^

Oxygen is essential to drive the riboflavin/UVA CXL process and in its absence crosslink formation is impaired.^13^ It has been postulated that reduced efficacy with accelerated CXL protocols is due to more rapid oxygen depletion compared to the more prolonged but less intense UVA exposure in SCXL.^14,15^ As oxygen can be restored to its normal tissue levels within 3 to 4 minutes of cessation of UVA radiation,^16^ it has been postulated that by pulsing the UV light, oxygen levels may be replenished so that the CXL process is no longer impaired.^17,18^ In addition to pulsing, some investigators have demonstrated that the efficacy of accelerated CXL protocols may be improved by increasing the UVA exposure time, and, hence, the overall cumulative dosage, by 30% to 40%.^19,20^ In this present study, we tested a number of these newer commercially available extended and pulsed accelerated protocols by investigating their resistance to enzymatic (pepsin) digestion.

All eyes treated in our study received an application of an isoosmolar riboflavin solution (containing 20% dextran) to the deepithelialized corneal surface. Consistent with previous studies,^21,28^ this resulted in a significant decrease in corneal thickness. The corneal thinning can be attributed primarily to the deturgescent effect of the dextran but also, possibly, to the presence of riboflavin,^26^ which has the effect of increasing the ionic strength of the applied solution and presumably further lowering the hydration of the cornea.^29^

Due to treatment-induced variations in corneal thickness and the swelling of the trephined corneal disks (in the posterior-anterior direction) during the first 24 hours of immersion in pepsin digest solution,^26^ corneal thickness measurements were considered to be an unreliable measure of the rate of enzymatic digestion. Instead, in this current study, daily measurement of the diameter of the anterior corneal surface and the dry weight of the undigested tissue after 13 days of digestion was performed. These measurements provided a more accurate assessment of the structural integrity of the anterior corneal stroma and the effective depth of CXL following each treatment variation.

The discovery that corneas treated with HCXL (5.4 J/cm^2^) had a lower residual mass after 13 days of digestion, and took less time to undergo complete digestion than SCXL-treated corneas, suggests a reduced CXL effect and a failure of the Bunsen–Roscoe law of reciprocity at higher UVA intensities. This supports the findings of biomechanical studies which have reported a reduced corneal stiffening effect with increasing UVA intensity up to 18mW,^30^ and a sudden decrease in efficacy with very high intensities greater than 45 mW/cm^2^.^31^ The failure of the Bunsen-Roscoe law of reciprocity in cases of very high intensity and short illumination time is not yet fully understood, but is thought to be caused, as discussed above, by insufficient oxygen availability inhibiting the CXL process.^13^ This hypothesis is supported by our findings of increased enzymatic resistance in p-HCXL treated eyes, where oxygen availability theoretically should be greater than in the nonpulsed HCXL treatments.

The precise photochemical mechanisms involved in riboflavin/UVA CXL are unknown. It has been postulated that the process commences under aerobic conditions with a brief type II photochemical reaction, in which sensitized photooxidation of stromal proteins occurs, mainly by their reaction with photochemically generated reactive oxygen species.^16^ As oxygen becomes depleted, after the initial 15 seconds of exposure to UVA, a type I photosensitizing mechanism then may predominate, in which radical ions are produced that can induce covalent CXL of stromal macromolecules.^16^ In SCXL, where UVA exposure occurs over 30 minutes, the oxygen concentration in the cornea may slowly increase, during the later stages of the treatment, to a level at which a type-II mechanism may once again begin.^16^

The enhanced enzymatic resistance we observed when the exposure time of the cornea to 30 mW UVA was increased from 3 to 4 minutes may be attributed to the increase in the total energy dose from 5.4 to 7.2 J/cm^2^, which allowed additional type I photochemical CXL to occur. It is unlikely that the increased CXL effect is due to the extended treatment providing additional time for the oxygen levels to be replenished to a level at which the type II CXL reaction could be restarted as, even when SCXL is performed, the oxygen concentration is thought only to reach sufficient levels in the latter half of the 30-minute treatment.^16^

Although corneas treated with HCXL and p-HCXL and a total energy dose of 7.2 J/cm^2^ persisted longer in enzyme digest solution than SCXL (5.4J/cm^2^)–treated corneas, the dry weight of the SCXL-treated corneas was higher when measured midway through the digestion process. This suggested that differences in the distribution of CXL may exist within the tissue. It can be postulated that CXL using a higher UVA intensity and a greater total energy dose results in superior CXL efficacy within the most anterior stromal layers and/or the mid-corneal region, resulting in longer overall digestion times. However, the depth of CXL may be shallower or there may be a more rapid decrease in the intensity of CXL as a function of depth, compared to SCXL, resulting in a reduced overall mass of crosslinked tissue. It is of interest that Brillouin microscopy studies of SCXL-treated corneas have shown that the intensity of CXL is depth-dependent, with the anterior stroma contributing the most to the increase in mechanical stiffness, and examination of the effect of varying UVA exposure time (0–30 minutes) has shown a dose-dependent tissue stiffening in the anterior third of the cornea.^32^

The clinical relevance of these findings is uncertain, as the precise amount and location of crosslinked tissue required to prevent keratoconic progression has yet to be determined. Clinical studies comparing CXL with accelerated CXL protocols are limited and somewhat conflicting. Tomita et al.,^33^ in a study comparing SCXL with 30 mW/cm^2^ for 3 minutes, found no differences in visual and topographic indices and similar demarcation line depths at 6 months, while Shetty et al.,^34^ in a randomized clinical study in 138 eyes with 12-month follow-up, found poorer refractive and tomographic outcomes with 30 mW/cm^2^ for 3 minutes. A small clinical trial involving 20 patients treated with either pulsed or continuous light HCXL showed keratoconus stability in both groups at 1 year follow-up, although the pulsed light treatment produced better functional outcomes and a deeper stromal penetration.^17^ Similarly, a retrospective assessment of 60 patients treated with HCXL found the demarcation line to be significantly deeper in patients treated with pulsed rather than continuous light.^35^ Clearly further, comparative clinical studies are required especially comparing the outcomes of p-HCXL with standard CXL, both of which appeared on this basis of this study to offer the best outcomes in terms of resistance to enzyme digestion.
